# Analysis on metabolic functions of rhizosphere microbial communities of *Pinus massoniana* provenances with different carbon storage by Biolog Eco microplates

**DOI:** 10.3389/fmicb.2024.1365111

**Published:** 2024-03-06

**Authors:** Zichen Huang, Yiyun Qin, Xin He, Mengyang Zhang, Xingyue Ren, Wenya Yu, Kongshu Ji

**Affiliations:** State Key Laboratory of Tree Genetics and Breeding, Key Open Laboratory of Forest Genetics and Gene Engineering of National Forestry and Grassland Administration, Co-Innovation Center for Sustainable Forestry in Southern China, Nanjing Forestry University, Nanjing, China

**Keywords:** *Pinus massoniana*, rhizosphere microorganisms, Biolog Eco, carbon source metabolism, functional diversity

## Abstract

**Introduction:**

Rhizosphere microorganisms are influenced by vegetation. Meanwhile, they respond to vegetation through their own changes, developing an interactive feedback system between microorganisms and vegetation. However, it is still unclear whether the functional diversity of rhizosphere soil microorganisms varies with different carbon storage levels and what factors affect the functional diversity of rhizosphere soil microorganisms.

**Methods:**

In this study, the Biolog-Eco microplate technique was used to analyze the metabolic diversity of carbon source of rhizosphere soil microorganisms from 6 Pinus massoniana provenances with three levels of high, medium and low carbon storage.

**Results:**

The results showed that the average well color development(AWCD) value of rhizosphere microorganisms was significantly positive correlated with carbon storage level of Pinus massoniana (*p* < 0.05). The AWCD value, Simpson and Shannon diversity of high carbon sequestrance provenances were 1.40 (144h incubation) 0.96 and 3.24, respectively, which were significantly higher (*p* < 0.05) than those of other P. massoniana provenances. The rhizosphere microbial AWCD, Shannon and Simpson diversity of the 6 provenances showed the same variation trend (SM>AY>QJ>SX>HF>SW). Similarly, microbial biomass carbon (MBC) content was positively correlated with carbon storage level, and there were significant differences among high, medium and low carbon storage provenances. The PCA results showed that the differences in the carbon source metabolism of rhizosphere microorganisms were mainly reflected in the utilization of amino acids, carboxylic acids and carbohydrates. Pearson correlation analysis showed that soil organic carbon (SOC), total nitrogen (TN) and pH were significantly correlated with rhizosphere AWCD (*p* < 0.05).

**Conclusion:**

Soil properties are important factors affecting rhizosphere microbial carbon source metabolism. The study confirmed that the microorganisms of high carbon storage provenances had relatively high carbon metabolic activity. Among them, the carbon metabolic activity of rhizosphere microorganisms of SM provenance was the highest, which was the preferred provenances in effective ecological service function.

## 1 Introduction

As the largest carbon reservoir in the terrestrial ecosystem, forests play a vital role in mitigating greenhouse gas emissions and slowing down global warming (Chen et al., 2022). Forest ecosystems effectively regulate CO_2_ accumulation by converting it into organic carbon through plant photosynthesis and sequestering it in plants or soil, a process known as forest carbon storage (Cai et al., 2023). According to statistics, the total global forest carbon storage is 662PgC, with approximately 45% stored in above-ground biomass carbon (Li et al., 2020). Recently, with the increasing problems of climate change and carbon emissions, forest carbon storage research has become a global priority. More and more studies have focused on the correlation between forest above-ground biological carbon storage and environmental factors such as climatic conditions (Labriere et al., 2023), soil moisture and temperature (Mercado et al., 2009), total carbon content (Jiao et al., 2016), and total nitrogen (TN) content (Heinrich et al., 2021).

*Pinus massoniana*, with its substantial forest carbon storage, has become a primary afforestation species in barren mountains in China due to its adaptability, wide planting range, and ease of survival. It is the most widely distributed species of *Pinus* in China (Yao Z. et al., 2023). The distribution area of *P. massoniana* covers 1.4 × 10^7^ hm^2^, and its total area and stock volume rank first among conifer species in China (Hu et al., 2022). Furthermore, the total carbon stock of *P. massoniana* forest is approximately 908.21 million m^3^, making it the third most abundant tree species in China after Chinese fir and poplar (Ji et al., 2022). In southern China, *P. massoniana* forests provide more significant ecological service benefits compared to other forest types, playing a key role in carbon sink ecosystems and subtropical forests in China (He et al., 2022). Studies have shown that there is a certain pattern of change in the average carbon storage of different *P. massoniana* families or provenances, with spatial distribution displaying regularity (Pan et al., 2017). Soil microorganisms are widely involved in various soil biochemical processes within forest ecosystems, such as organic matter decomposition, mineralization, and degradation (Zhou et al., 2023). They play a pivotal role in soil nutrient cycling and biogeochemical processes (Delgado-Baquerizo et al., 2016). Previous research has demonstrated significant differences in rhizosphere soil microbial community structure among *P. massoniana* provenances with different levels of carbon storage, exhibiting certain correlations with the organic matter content of the rhizosphere soil (Huang et al., 2023).

The rhizosphere refers to the surface of roots and the microzone of the soil layer in close proximity to the roots. Rhizosphere soil is defined as the soil adhering to the roots within a thickness of approximately 1 mm, serving as an important site for communication between plants and rhizosphere microorganisms (Schweinsberg Mickan et al., 2012). Rhizosphere microorganisms are not only influenced by plants (Lucas Borja et al., 2012) but also establish an interactive feedback system with plants through their own modifications (Zinn et al., 2005). Within the rhizosphere, microorganisms are involved in nutrient cycling and metabolic processes. They indirectly impact plant growth and health by improving soil organic matter content (Delgado-Baquerizo et al., 2016; Deng et al., 2019). Microbial metabolic function diversity can be used to assess soil processes and ecological functions (Xia et al., 2022). The functional diversity of soil microorganisms is affected by various factors such as vegetation cover, elevation gradient, and soil physical and chemical properties (Wei et al., 2009; Burton et al., 2010; Ibell et al., 2010). Researchers have found that land cover conversion significantly influences surface soil carbon and nitrogen storage as well as soil microbial functional diversity in alpine meadows on the Qinghai-Tibet (Zhu et al., 2015). Additionally, different altitude gradients have significant effects on the metabolic diversity of microbial communities in alpine meadows (Wang et al., 2018). Forest ecosystems play a pivotal role in maintaining the functional diversity of soil microorganisms. There are differences in the maintenance of functional diversity among soil microorganisms across different forest stand types (Yao Z. W. et al., 2023). However, there have been limited studies on the effects of plant carbon storage levels on the functional diversity of soil microorganisms.

The main methods for assessing the structural composition and functional diversity of soil microbial communities are plate colony counting, cell morphology, and the 16S/ITS amplification technique (Ge et al., 2018). However, these methods often have disadvantages, including complex operation, time-consuming analysis, and poor repeatability. Researchers have developed the Biolog Eco microplate technology as a simpler and faster method to evaluate microbial metabolic functional diversity (Feigl et al., 2017). The Biolog Eco microplates can characterize microbial functions based on metabolic activity and were specifically designed for analyzing bacterial communities in environmental samples (Li et al., 2021). They provide comprehensive information on the function of six types of soil microorganisms, including 31 carbon sources, for microbial community analysis (Zhang et al., 2023). Studies have shown that plant diversity (Zhu et al., 2015) and forest type are closely linked to soil microbial functional diversity (Yao Z. W. et al., 2023), influencing soil microbial structure, activity, and biomass. Although these studies have focused on the correlation between above-ground vegetation and soil microorganisms, few have investigated the impact of woody plants with different levels of carbon storage on soil microbial functional diversity using the Biolog Eco microplates.

In this study, we compared the functional diversity of rhizosphere soil microorganisms at high, medium, and low carbon storage levels of *P. massoniana* provenances. Our research addresses two questions: (1) Is there a significant difference in the microbial functional diversity of rhizosphere soil among *P. massoniana* provenances at different carbon storage levels? and (2) What are the key factors that influence microbial functional diversity in the rhizosphere soil of *P. massoniana* plantations? Analyzing the microbial functional diversity in the rhizosphere soil of *P. massoniana* provenances with different carbon storage levels and identifying its influencing factors hold significant importance for the research on the carbon cycle system of *P. massoniana* plantation forests.

## 2 Materials and methods

### 2.1 Study site and experimental design

Our sampling location was the state-owned forest seed farm in Yu‘an District, Lu‘an City, Anhui Province, China. The farm is situated at an elevation between 80 and 100 m, with slopes ranging from 5°to 10°. The region experiences a north subtropical monsoon climate, with an annual rainfall of 1239.8 mm and an average annual temperature of 15°C. The soil predominantly consists of clay disks of yellow-brown soil derived from loess, with a slightly acidic pH. The soil depth is considerable, ranging mainly from 70 to 150 cm.

The geographical provenance test forest of *P. massoniana* was established in 1981, covering a total area of 11.67 hm^2^. It consists of 64 provenances, representing geographical locations in 14 provinces and autonomous regions in China. Each provenance was arranged in a completely randomized block experiment, divided into six cell groups separated by trails. There were 64 provenances in each plot, with 8 provenances in each row and column and 9 duplicate provenances within each plot, with a plant spacing of 1 m x 1 m. The experimental forest has not undergone thinning for the past 41 years. Due to insect pests, diseases, and growth competition among individual trees, some provenances did not meet the requirements for statistical analysis. Biomass estimation for different provenances was carried out using a binary model based on diameter at breast height (DBH) and tree height measurements (Hu et al., 2022) according to the Chinese Forestry Industry Standard (LY/T2263-2014). We multiplied the biomass of different organs estimated by the model by the corresponding carbon content coefficient (Zeng and Xiao, 2011). The carbon storage of each organ was obtained. The carbon storage of each plant from various sources was obtained by summing. According to the order of carbon storage from high to low, six provenances representing high, medium, and low carbon storage levels of *P. massoniana* were selected: AnYuan (AY) and SanMing (SM) for high carbon storage, ShaoWu (SW) and QingJiang (QJ) for medium carbon storage, and ShengXian (SX) and HeFeng (HF) for low carbon storage (Supplementary Table S1).

### 2.2 Soil sampling

For each *P. massoniana* provenance, we selected three trees with DBH and height measurements that were close to the mean value, serving as three replicates. A total of three 20-cm sections were taken in three directions (120°) from each tree, allowing for multiple transverse sections of thin roots with varying diameters. We specifically focused on fine roots with a diameter smaller than 2 mm and collected the rhizosphere soil adhering to their surfaces. The rhizosphere soil mixture of the three sections is mixed as a repetition. The collected soil was divided into two parts. One part was used for the determination of soil properties, while the other part was used for the assessment of soil microbial biomass carbon (MBC), nitrogen content, and the Biolog Eco microplate analysis.

### 2.3 Soil properties and microbial biomass

We used a glass composite electrode to determine the pH value with a ratio of water to soil of 1:2.5. Soil organic carbon (SOC) and TN were determined by an external heating method with potassium dichromate (Benbi, 2018) and a flow analyzer (Bunch and Bernot, 2012), respectively. Soil MBC and microbial biomass nitrogen (MBN) were determined by chloroform fumigation (Setia et al., 2012).

### 2.4 Biolog Eco microplate analysis

To analyze the differential microbial utilization of carbon sources, we used the Biolog Eco microplate method (Garland and Mills, 1991). The information on 31 carbon sources is shown in Supplementary Figure S1. A volume of 45 mL of sterile deionized water was added to a 100-ml sterilized triangle bottle containing 5 g of wet soil. The bottle was sealed and shaken at room temperature for 30 min. Next, 1 ml of soil suspension was transferred to a sterile centrifuge tube using a pipette and centrifuged at 8,000 rpm for 20 min. The supernatant was then discarded. A volume of 1 ml of sterile saline was added and thoroughly mixed. The tube was centrifuged at 8,000 rpm for 20 min. We added 1.2 ml of sterile normal saline after discarding the supernatant and thoroughly mixed it. Then, we centrifuged it at 2000 rpm for 1 min. Finally, 0.8 ml of the supernatant was transferred to a sterile test tube containing 20 ml of normal saline and mixed well. This mixture served as the reaction liquid. The Biolog Eco microplate was preheated to 25°C before use. A pipette was used to take 150 μl of the diluent into each well, adding double steaming water as a control. The microplate was then incubated at 28°C. At time intervals of 0, 24, 48, 72, 96, 120, 144, 168, 192, 216, and 240 h, the absorption value was measured at 590 nm.

The AWCD and its change over time serve as indicators of overall microbial metabolic activity. It is generally recognized that samples with a higher AWCD value exhibit greater carbon source metabolic activity, indicating higher microbial abundance characteristics. AWCD is calculated as follows:


$$\mathrm{AWCD}=\left[\sum \left(\mathrm{Ci}-R\right)\right]/31\text{.}$$

Ci is the absorption value of the well, except for the control well. R is the absorption value of the control well.

The utilization values (OD595) of 31 carbon sources formed a multivariate vector of the microbial metabolic characteristics of each sample. Shannon diversity, richness index, and Simpson diversity were further used to assess the diversity of bacterial community metabolic functions. The diversity function in the R language’s vegan package was used to calculate the diversity index. SPSS 19.0 software was used to carry out principal component analysis (PCA). The remaining charts were created with Origin2022.

## 3 Results

### 3.1 Soil properties

The SOC content exhibited significant differences among the six provenances (*p* < 0.05), with the order of SM > AY > QJ> > SW > HF > SX (Table 1). SM and AY, which were high carbon storage provenances, had significantly higher SOC content compared to other provenances. Meanwhile, the SOC content of HF provenance was significantly higher than that of SX provenance. Similarly, SM showed the highest TN content, followed by QJ. The TN content of SM and QJ was significantly higher than that of other provenances (*p* < 0.05). The descending order of TN content was SM > QJ > AY > SX > HF > SW. The TN content of SW, HF, and SX provenances was significantly lower than that of other provenances (p < 0.05). The pH values demonstrated significant variation among the six provenances. The descending order of pH values was SX > HF > QJ > SW > AY > SM. The trend observed was low carbon storage > medium carbon storage > high carbon storage. The pH value of the SM provenance was significantly lower than that of other provenances (*p* < 0.05).

**Table 1:** Rhizosphere soil properties of *P. massoniana* provenances.

	MBC	MBN	MBC/MBN	pH	SOC	TN
SM	335.05 ± 23.46a	15.63 ± 0.65ab	21.62 ± 2.38ab	4.61 ± 0.03c	27.59 ± 1.05a	2.61 ± 0.08a
AY	284.18 ± 35.31b	18.80 ± 1.89a	15.99 ± 4.34b	4.74 ± 0.05bc	26.51 ± 0.63a	1.68 ± 0.09b
SW	234.56 ± 16.98c	7.90 ± 1.44c	32.96 ± 9.28a	4.81 ± 0.07b	16.49 ± 0.10b	1.23 ± 0.05c
QJ	198.92 ± 13.70 cd	15.56 ± 1.28ab	12.81 ± 0.22b	4.85 ± 0.02b	25.11 ± 2.01a	2.59 ± 0.19a
HF	225.89 ± 36.17 cd	13.68 ± 2.85b	16.38 ± 2.14b	4.89 ± 0.05b	12.29 ± 0.41c	1.25 ± 0.02c
SX	175.90 ± 7.82d	12.81 ± 1.92b	14.23 ± 1.88b	5.09 ± 0.01a	9.00 ± 0.49d	1.29 ± 0.12c

AY, AnYuan provenance; SM, SanMing provenance; SW, ShaoWu provenance; QJ, QingJiang provenance; HF, HeFeng provenance; SX, Zhejiang Shengxian provenance; different letters indicate significant differences among provenances using a one-way ANOVA (LSD, *P* < 0.05).

There were significant differences in MBC content among the six provenances (Table 1). The MBC content followed the order of SM > AY > SW > HF > QJ > SX from high to low. The MBC content of high carbon storage provenances, AY and SM, was significantly higher than that of the medium and low carbon storage provenances (*p* < 0.05). Conversely, the MBC content of SX was significantly lower compared to other provenances (*p* < 0.05). The MBN content of the AY provenance (high carbon storage) was significantly higher than that of the medium and low carbon storage provenances (SW, HF, and SX), followed by the SM and QJ provenances (*p* < 0.05). Furthermore, the MBN content of SW was significantly lower than that of HF and SX (*p* < 0.05). The descending order of MBC/MBN was SM > QJ > AY > SW > SX > HF. The MBC/MBN ratio of SW provenances was significantly higher than that of other provenances (*p* < 0.05).

### 3.2 Functional diversity of soil microorganisms

**Figure 1:**
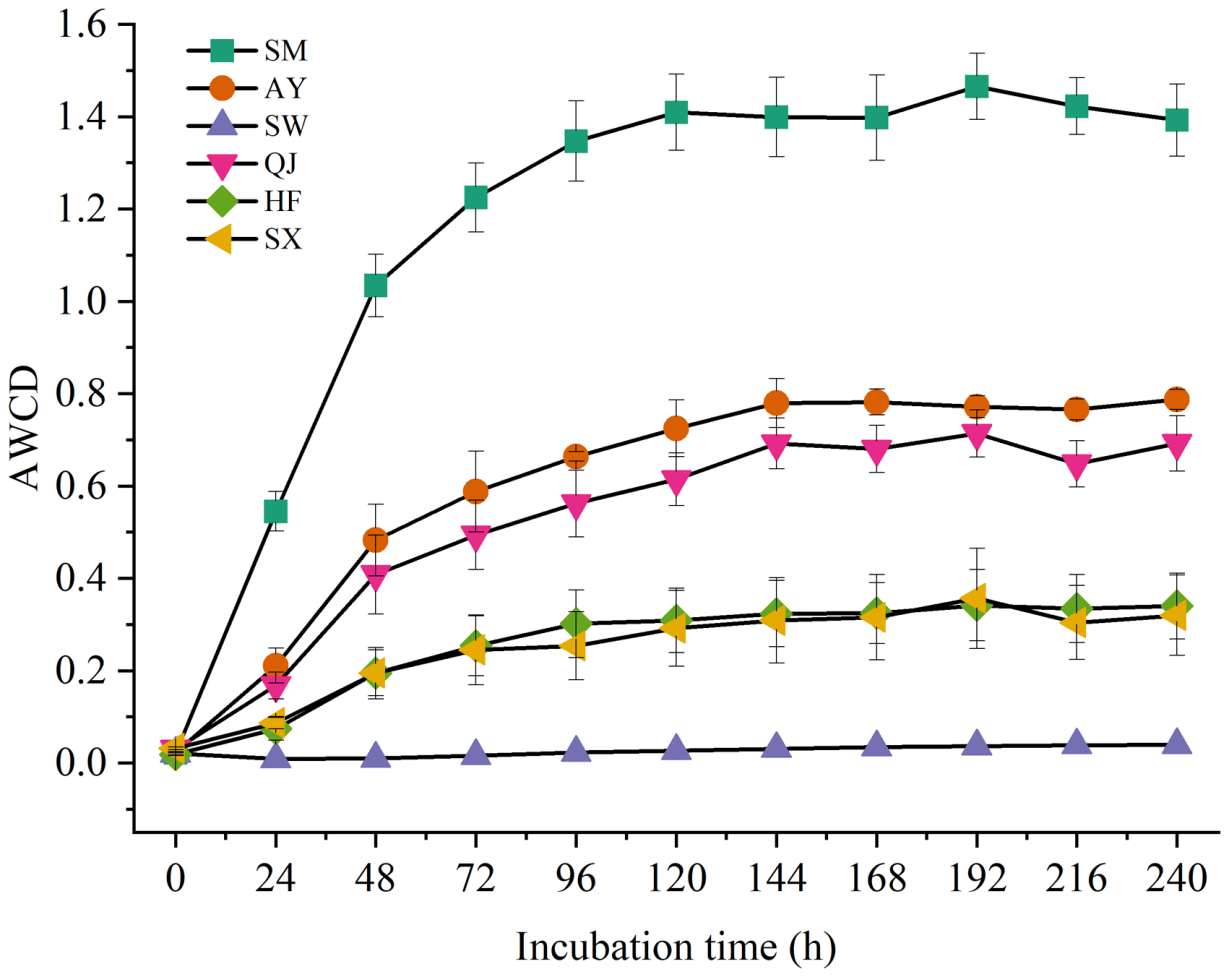
AWCD of carbon sources in the rhizosphere soil microbial communities of *P. massoniana* provenances within the incubation time.

The AWCDs of rhizosphere soil microorganisms from different *P. massoniana* provenances with varying carbon storage levels are shown in Figure 1. The results showed that AWCD values among the rhizosphere microorganisms of different *P. massoniana* provenances were significantly different (*p* < 0.05). Furthermore, the AWCD values of all rhizosphere microorganisms exhibited a distinct hysteresis effect after 24 h of culture. Then, with the increase in culture time, the AWCD value gradually increased. Notably, the AWCD dynamics of SM were significantly higher than those of other provenances (*p* < 0.05), followed by AY and QJ provenance. The results indicated that rhizosphere microorganisms from high carbon storage provenances exhibited the highest metabolic activity toward soil carbon sources. At the end of the culture period, the AWCD values for SM and AY were 1.39 and 0.78, respectively. Compared with SM and AY, the AWCD values for medium and low carbon storage provenances increased at a slower rate. The metabolic rate of SW was the lowest, with AWCD values reaching only 0.03 after 240 h of culture.

**Figure 2:**
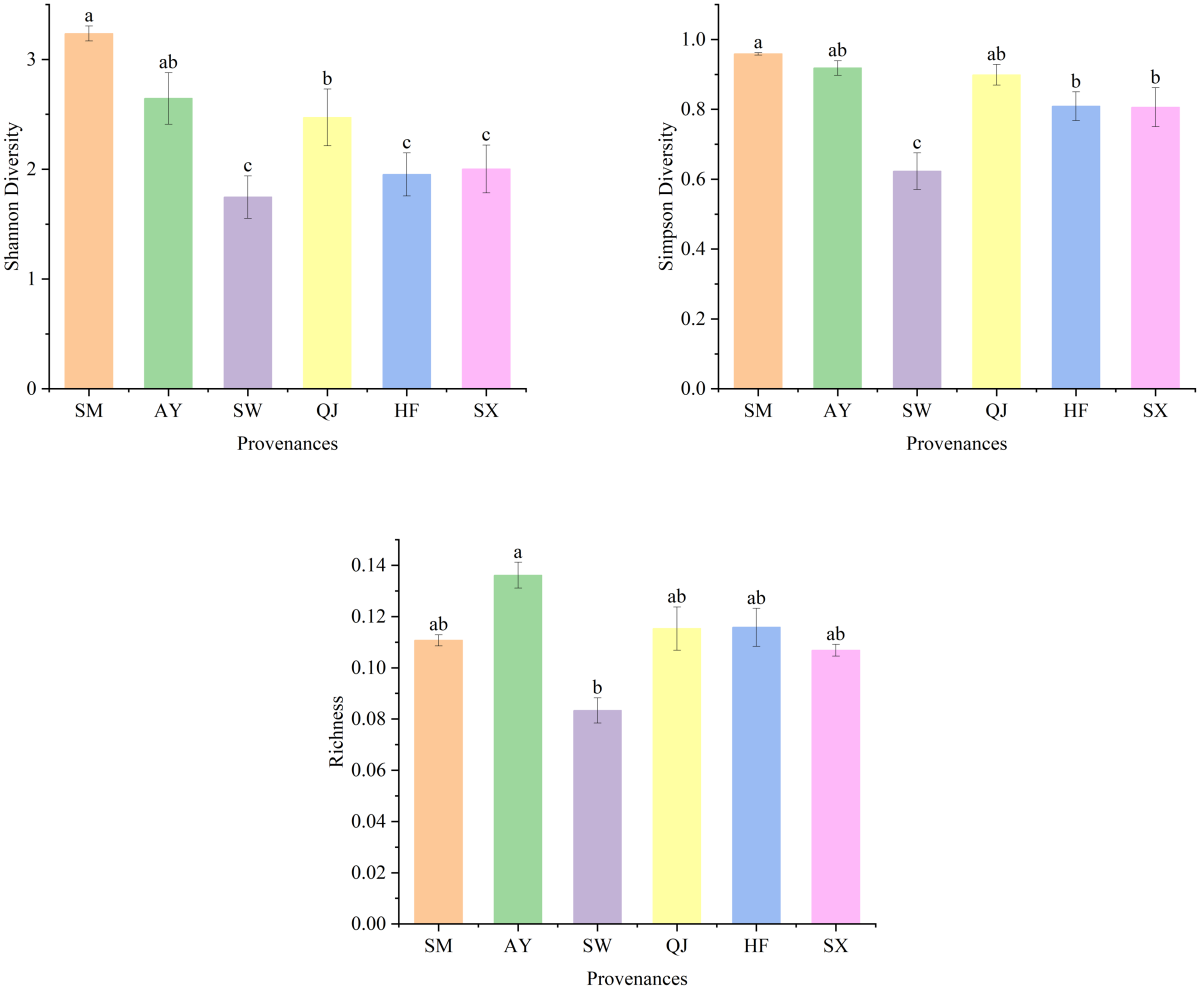
Shannon diversity, Simpson diversity, and richness index of soil microorganisms in different *P. massoniana* provenances. Data are means ± standard errors, and different lower-case letters indicate significant differences among provenances (*p* < 0.05).

The metabolic function diversity of rhizosphere microorganisms was evaluated using the Shannon diversity index (H), Simpson diversity index (D), and richness indexes after incubation for 192 h (Figure 2). Higher diversity index values indicate a greater variety of carbon sources available to microbes (Xia et al., 2015). The Shannon diversity index exhibited the following descending order: SM > AY > QJ > SX > HF > SW. Among them, the Shannon diversity of SM provenances was significantly higher than that of medium and low carbon storage provenances, including QJ, SW, HF, and SX (*p* < 0.05). Overall, the Shannon diversity displayed the trend of high carbon storage>medium carbon storage > low carbon storage. The Simpson diversity index followed the order of SM > AY > QJ > HF > SX > SW from high to low. The Simpson diversity of high carbon storage provenances SM and AY and medium carbon storage provenance QJ was significantly higher than that of other provenances. Richness showed the descending order of AY > HF > QJ > SM > SX > SW. The richness of AY was significantly higher than SW (*p* < 0.05).

### 3.3 PCA of carbon source metabolization

**Figure 3:**
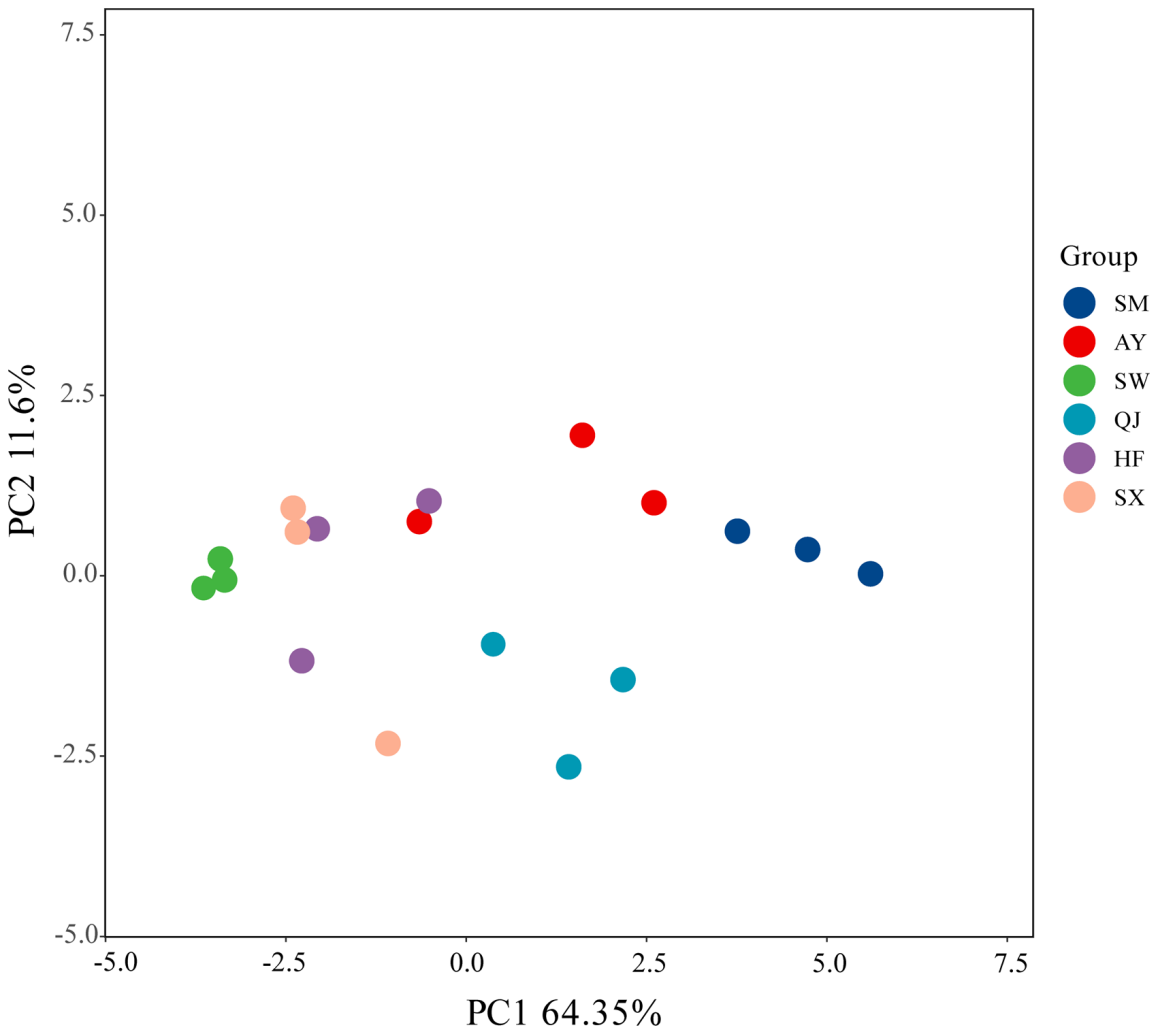
Principal component analysis (PCA) for the carbon source utilization of soil microbial communities in different *P. massoniana* provenances.

PCA was conducted on absorbance values incubated for 144 h (Figure 3). The first and second principal components accounted for variance contribution rates of 64.35 and 11.60%, respectively. Figure 3 shows the distribution range of the six provenances, which were differentiated along the PC1 axis and PC2 axis. SM, AY, and QJ were located in the direction of PC1, while SW, HF, and SX were positioned in the negative direction of the PC1 axis. The distribution of high, medium, and low carbon storage provenances in different quadrants indicated that there were significant differences in the utilization of carbon sources among the various provenances.

Table 2 shows the loading scores of the 31 carbon sources in the first two principal components. Larger loading scores indicate a stronger influence of the carbon source on the principal component. Carbon sources with an absolute value greater than 0.6 are considered the main types of carbon sources utilized by the soil microbial community (Yao Z. W. et al., 2023). As can be seen from the results in Table 2, there are 10 types of carbon sources that primarily affect PC1. These sources include three types of amino acids, three types of carboxylic acids, one type of amine, two types of polymers, and one type of phenolic acid. Among them, 4-hydroxy benzoic acid is the carbon source most related to PC1 (load score = 0.881), followed by Tween 40 (0.870) and γ-hydroxy butyric acid (0.861). There are seven carbon sources primarily influenced by PC2, consisting of six types of carbohydrates and one type of amino acid. D-Cellobiose had the strongest connection with PC2 (load score = 0.857), followed by D,L-α-glycerol phosphate (0.821) and glucose-1-phosphate (0.780).

**Table 2:** Loading scores of 31 carbon sources on PC1 and PC2.

Type of carbon source	Plate number	Kind of carbon source	PC1	PC2
Amino acids	A4	L-Arginine	**0.743**	−0.033
	B4	L-Asparagine	**0.655**	0.459
	C4	L-Phenylalanine	**0.702**	0.544
	D4	L-Serine	0.577	**0.659**
	E4	L-Threonine	0.279	0.393
	F4	Glycyl-L-glutamic acid	0.259	**0.639**
Carbohydrates	G1	D-Cellobiose	0.222	**0.857**
	H1	α-D-Lactose	0.042	0.56
	A2	Methyl-D-glucoside	0.418	0.419
	B2	D-Xylose	0.238	0.293
	C2	i-Erythritol	0.199	0.407
	D2	D-Mannitol	0.279	**0.711**
	E2	N-Acetyl-D-glucosamine	0.202	**0.696**
	F2	D-Glucosaminic acid	0.421	0.158
	G2	Glucose-1-phosphate	0.227	**0.78**
	H2	D,L-α-Glycerol phosphate	0.225	**0.821**
	A3	D-Galactonic acid lactone	0.267	**0.757**
	B3	D-Galacturonic acid	0.309	0.573
Carboxylic acids	E3	γ-Hydroxy butyric acid	**0.861**	0.13
	F3	Itaconic acid	0.125	0.265
	G3	α-Keto butyric acid	0.069	0.131
	H3	D-Malic acid	**0.78**	0.233
	B1	Pyruvic acid methyl ester	**0.702**	0.522
Amines	G4	Phenylethylamine	0.149	0.361
	H4	Putrescine	**0.661**	0.51
Polymers	C1	Tween 40	**0.87**	0.204
	D1	Tween 80	**0.698**	0.33
	E1	α-Cyclodextrin	0.304	0.071
	F1	Glycogen	0.342	0.449
Phenolic acid	C3	2-Hydroxy benzoic acid	0.205	0.183
	D3	4-Hydroxy benzoic acid	**0.881**	0.113

Loading scores with absolute values greater than 0.6 are shown in bold.

### 3.4 Metabolic utilization of biochemical classification substrates

**Figure 4:**
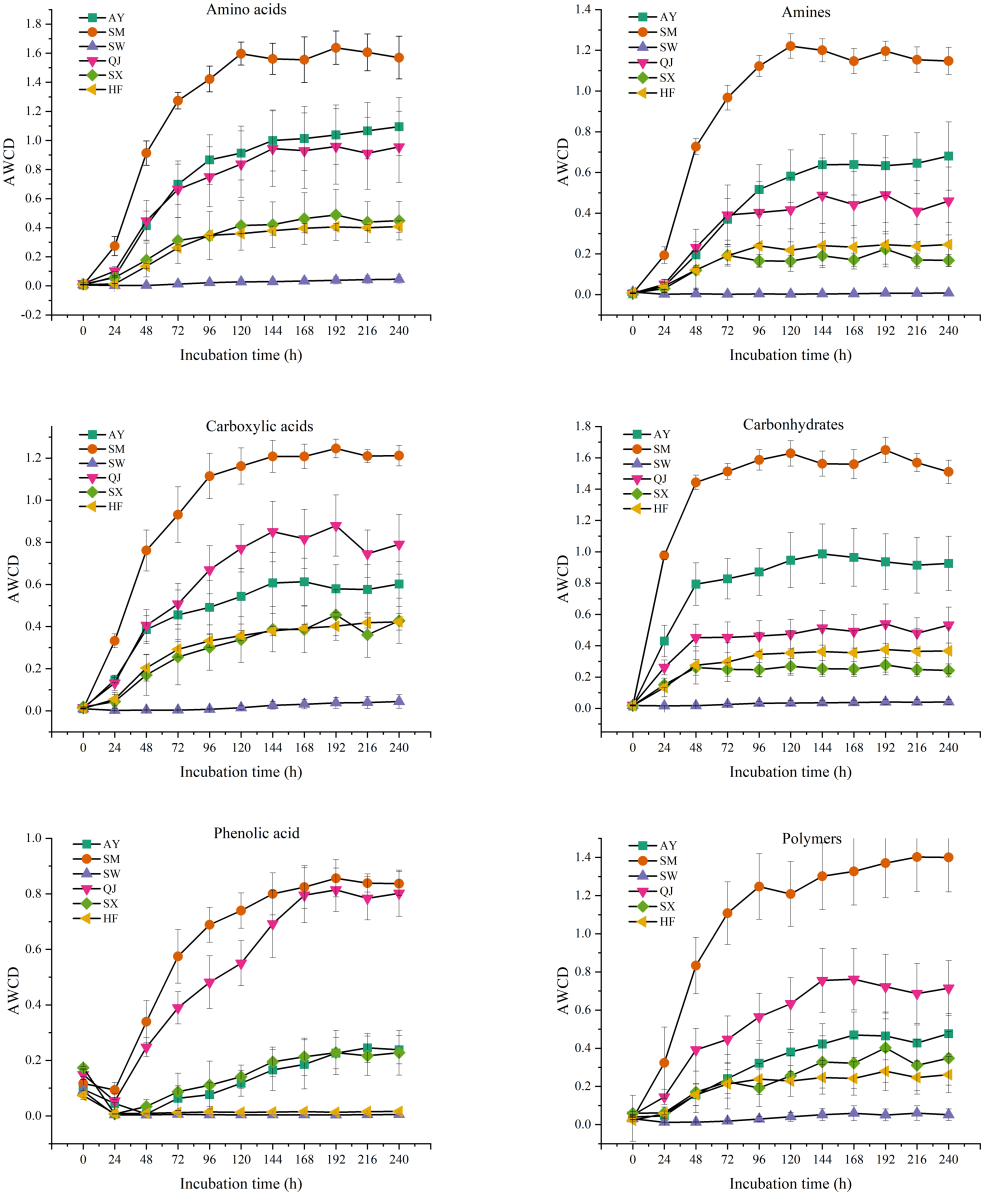
Average well-color development of six types of carbon source microbial communities from different *P. massoniana* provenances after inoculation for 240 h, including amines, amino acids, carboxylic acids, carbohydrates, phenolic acid, and polymers.

According to their biochemical properties, the 31 carbon source substrates in the Biolog Eco microporous plates can be divided into six types: amines, amino acids, carbohydrates, carboxylic acids, phenolic acids, and polymers (Table 2). The rhizosphere microbial communities of *P. massoniana* provenances have different capacities for utilizing the six carbon sources. We summarized the AWCD results for the six types of carbon sources (Figure 4). The results showed significant differences in the utilization of carbohydrates among different *P. massoniana* provenances. The utilization capacity of the other five carbon sources, except for polymers, was significantly higher for SM than for other provenances (*p* < 0.05). Among them, AY had the second highest utilization efficiency for amino acids, amines, and carbohydrates, followed by SM. QJ exhibited the second highest utilization rate for carboxylic acids. There was no significant difference in the utilization efficiency of the mentioned four carbon sources for the two low carbon storage provenances (HF and SX). Both of them were lower than the AY and SM provenances. SW had the lowest utilization efficiency of the six carbon sources. Compared with other carbon sources, the utilization rate of polymers varies greatly. AY had the highest utilization rate for polymers, followed by QJ, while SM ranked third.

### 3.5 Correlation analysis between the utilization of C sources and environmental factors

**Figure 5:**
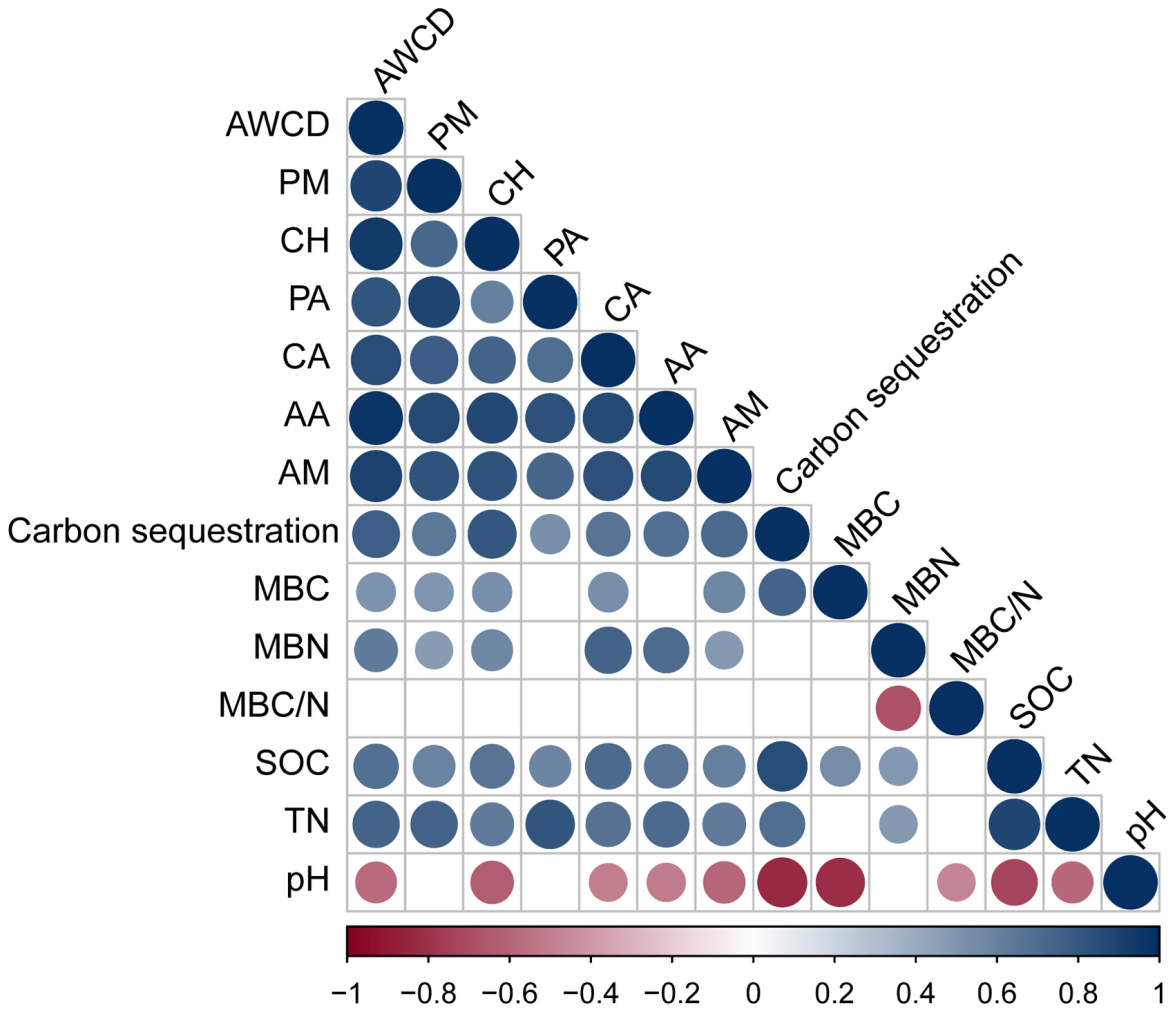
Correlation analysis between the utilization of carbon sources, carbon storage levels, and soil properties. Those with significant correlations are circled. If there is no significant correlation, it is blank. Soil properties: pH, soil pH; SOC, soil organic carbon; TN, total nitrogen; MBC, microbial biomass carbon; MBN, microbial biomass nitrogen; MBC/N, microbial biomass carbon/nitrogen. Six carbon source types: CH, carbohydrates; CA, carboxylic acids; AA, amino acids; PM, polymers; PA, phenolic compounds; AM, amines.

We performed Pearson’s correlation analysis to examine the correlation between rhizosphere microbial carbon source utilization and carbon storage levels, as well as the soil properties of *P. massoniana* provenances (Figure 5). The results revealed a significant positive correlation between the utilization rates of each carbon source (PM, CH, PA, CA, AA, and AM) (*p* < 0.05). The rates were significantly positively correlated with SOC and TN (*p* < 0.05). The carbon storage levels of the provenances were also positively correlated with the utilization rates of these carbon sources (*p* < 0.05). Moreover, the soil MBC content had positive correlations with the utilization rates of PM, CH, and AM. Soil pH had a negative influence on the utilization rates of CH, AA, and AM. MBN content displayed positive correlations with the utilization rates of PM, CH, CA, AA, and AM (*p* < 0.05). In addition, the MBC content was significantly positively correlated with the SOC content and negatively correlated with the pH value (*p* < 0.05). The MBN content was significantly positively correlated with SOC and TN contents (*p* < 0.05). Moreover, MBC/N was significantly negatively correlated with the pH value (*p* < 0.05).

## 4 Discussion

### 4.1 Effects of different *Pinus massoniana* provenances on the contents of MBC, MBN, and MBC/N in rhizosphere soil

In this study, the MBC content of different *P. massoniana* provenances significantly varied. Studies have shown that different pine genotypes have differences in productivity resource allocation, leading to variations in underground root turnover rate and fine-root standing crop, which can impact microbial biomass (microbial activity) (Tyree et al., 2014). The MBC content of SM provenances was significantly higher than that of other provenances in this study. Therefore, we speculate that SM provenances possess a higher turnover rate and a standing crop of fine roots. In this study, there was a significant positive correlation between MBC content and carbon storage of *P. massoniana* (*p* < 0.05). Plants sequester carbon through photosynthesis, and the products of photosynthesis are transported into the soil as root exudates and litter (Terrer et al., 2019). Provenances with high carbon storage exhibit greater input of soil carbon into the rhizosphere soil, thereby increasing the MBC content. In this study, compared to other provenances, SW provenances demonstrated a significant reduction in MBN content, while MBC/N was relatively high. MBN represents the most active organic nitrogen component in soil, reflecting soil microbial nitrogen retention and mineralization dynamics (Dou et al., 2023). The soil MBC/N is also a reflection of the nitrogen supply capacity of the soil. Soil nitrogen shows higher bioavailability when the microbial carbon-to-nitrogen ratio is low (Fu et al., 2012). Therefore, we concluded that SW provenances exhibit lower rates of rhizosphere nitrogen mineralization. As a medium carbon storage provenance, the content of MBN and TN in the QJ provenance was similar to that of the SM provenance from the high carbon storage provenance. The MBC/N was lower in the QJ provenance, which suggested that the rhizosphere soil of the QJ provenance exhibited higher nitrogen bioavailability. This difference is likely attributed to changes in root exudation as well as the rate of fine-root turnover (Tyree et al., 2014).

### 4.2 Soil microbial functional diversity

The AWCD values of the high carbon storage provenances SM and AY were significantly higher than those of other provenances at each period of the 240 h culture in this study. These differences can be attributed to plant species, root exudates, litter decomposition, and plant residues (Yao Z. W. et al., 2023). Both Shannon diversity and Simpson diversity of rhizosphere microorganisms with high carbon storage provenances (SM and AY) were significantly higher than those from medium and low carbon storage (*p* < 0.05). The results showed that the microbial functional diversity of rhizosphere soil in high carbon storage provenances was significantly higher than that in medium and low carbon storage provenances. Rhizosphere microorganisms from high carbon storage provenance exhibited a greater ability to utilize various carbon sources (Yao Z. W. et al., 2023). On the whole, high carbon storage provenances exhibited enhanced forest ecological functions. Plant root secretions provide diverse nutrients to rhizosphere microorganisms, thereby improving soil microbial metabolic activity and increasing the utilization of carbon sources by soil microorganisms (Yao Z. W. et al., 2023). Studies have shown that the amount of carbohydrates secreted by plants into the rhizosphere soil positively correlates with the ability of rhizosphere microorganisms to utilize carbon substrates (Wu et al., 2014). In this study, high carbon storage provenances exhibited a high level of rhizosphere microbial carbon source metabolism, indicating that within the *P. massoniana* forest ecosystem, higher carbon storage levels corresponded to greater soil microbial functional diversity. In addition, the microbial carbon source metabolic diversity was also influenced by provenance differences. Variations in rhizosphere exudates among different provenances can lead to diversification in substrate utilization by rhizosphere microorganisms, thereby affecting their functional diversity and community structure (Doornbos et al., 2012). It has been reported that rhizosphere soil microbial diversity differs among plants with different genotypes (Kaiser et al., 2001). Our study showed that the functional diversity of rhizosphere soil microorganisms was significantly different among *P. massoniana* provenances with varying carbon storage levels. The microbial functional diversity of high carbon storage provenances was significantly higher than that of medium and low carbon storage provenances. Among them, the soil microbial function diversity and MBC of the SM provenance were significantly higher than in other provenances. Thus, the SM provenance is favored for its effective ecological service function. The results of PCA analysis showed that the six *P. massoniana* provenances were positioned in different quadrants, indicating that there were differences in their utilization of different carbon sources (Yao Z. W. et al., 2023). Amino acids, carbohydrates, and carboxylic acids were the key carbon sources that distinguished the metabolic characteristics of soil microbial communities. In other words, the differences in carbon source metabolism of rhizosphere microorganisms among different *P. massoniana* provenances primarily involved the utilization of amino acids, carbohydrates, and carboxylic acids.

### 4.3 Environmental regulation mechanism

Previous studies have shown that different genotypes of pine affect rhizosphere microbial activity (Tyree et al., 2014). However, the effects of different carbon storage genotypes (provenances) on the metabolic capacity of rhizosphere microorganisms have not been reported. In this study, the carbon storage of *P. massoniana* provenances was significantly positively correlated with the total microbial carbon source AWCD and the AWCD of each carbon source species (*p* < 0.05). This indicates a significant positive correlation between the carbon storage function of *P. massoniana* and the metabolic capacity of microbial carbon sources. Furthermore, an increase in rhizosphere organic carbon input had a positive effect on the metabolic capacity of soil microbial carbon sources (Xia et al., 2015). Soil physical and chemical properties and nutrients are widely recognized as critical drivers of soil bacterial communities (Nguyen et al., 2018; Rath et al., 2019; Yang et al., 2020). In this study, the rhizosphere SOC and TN contents of high carbon storage provenances were generally high, providing necessary nutrients and energy for soil microorganisms and promoting microbial activity. In addition, the pH value was significantly negatively correlated with the AWCD of CA, CH, PM, PA, and AM. These results indicate that pH value is another key factor affecting the carbon source metabolism ability of rhizosphere soil microorganisms in *P. massoniana* plantations. Multiple studies have demonstrated the influence of soil pH on microbial communities’ structure and distribution. For example, the study by Liu et al. (2014) showed that the composition and diversity of the bacterial community were significantly correlated with soil pH in black soil farmlands in northeast China. The rhizosphere soil of *P. massoniana* forest tends to be weakly acidic. As the carbon storage of *P. massoniana* provenances increases, the rhizosphere soil pH value decreases. Weakly acidic conditions can stimulate the growth of certain eosinophilic bacteria (Wang et al., 2014). The bacteria adapted to the long-term weak acidic conditions through their own regulatory system. Under the dual influence of organic matter and pH value, rhizosphere microorganisms from different *P. massoniana* provenances performed different metabolic characteristics (Liu et al., 2017).

In this study, there was a significant positive correlation between soil MBC and the metabolic diversity of microbial carbon sources. MBC content is an important indicator of soil microbial activity (Mendoza et al., 2020). A higher MBC content indicates greater microbial activity (Waldrop and Firestone, 2004). Accordingly, the metabolic activity of carbon sources also increases. Yao et al. also came to a similar conclusion that soil microbial biomass is an important indicator of the metabolic activity of microbial communities (Yao Z. W. et al., 2023). This relationship is primarily influenced by litter quantity and quality, as well as differences in fine-root turnover among different provenances (Brown and Markewitz, 2018). The diversity index of the SM provenance is significantly higher than that of other provenances, suggesting a relatively high rate of fine-root turnover. The results of the study confirmed that *P. massoniana* provenances with high carbon storage had a higher metabolic level in the rhizosphere microorganism, indicating their superior ability to maintain the diversity of the soil microbiotic community (Yao Z. W. et al., 2023). Among these provenances, the SM provenances showed a higher level of rhizosphere microbial metabolism compared to others, making it a preferable choice for establishing high carbon sequestration *P. massoniana* plantations in the future. These findings hold certain guiding significance for the scientific management of carbon storage forestry.

## 5 Conclusion

In this study, the Biolog Eco microplate method was used to study the rhizosphere microorganisms of different *P. massoniana* provenances. The results showed that there were significant differences in the carbon metabolism of rhizosphere microorganisms among the various *P. massoniana* provenances. Carbon metabolic activity increased with an increase in carbon storage levels, with the SM provenance exhibiting the highest carbon metabolic activity. The differences in carbon source metabolism among the rhizosphere microorganisms of different *P. massoniana* provenances were primarily reflected in the utilization of amino acids, carboxylic acids, and carbohydrates. The soil environmental factors that contributed to the variation in carbon metabolism utilization included pH, SOC, and TN.

The results of the study confirmed that *P. massoniana* provenances with high carbon storage had a higher metabolic level in the rhizosphere microorganism. The SM provenance is identified as a favorable provenance for maintaining soil microbial functional diversity in the *P. massoniana* ecosystem. The Biolog Eco microplate method can provide a fast and accurate assessment of the metabolic activity of the rhizosphere microbial community in *P. massoniana*. However, this method does have some limitations as it is only suitable for culturable bacterial communities, potentially missing other specialized functional microbiota. Therefore, to further explore the rhizosphere microbial community structure of different *P. massoniana* provenances, it will be necessary to combine molecular biology methods to examine the community structure, species richness, molecular ecological network, and other relevant factors.

## Data availability statement

The raw data supporting the conclusions of this article will be made available by the authors, without undue reservation.

## Author contributions

ZH: Conceptualization, Investigation, Software, Writing – original draft, Writing – review & editing. YQ: Conceptualization, Investigation, Methodology, Software, Writing – review & editing. XH: Data curation, Investigation, Methodology, Writing – review & editing. MZ: Data curation, Investigation, Methodology, Software, Writing – review & editing. XR: Investigation, Software, Visualization, Writing – review & editing. WY: Formal analysis, Software, Visualization, Writing – review & editing. KJ: Funding acquisition, Investigation, Writing – review & editing.

## References

[ref1] Benbi D. K. (2018). Evaluation of a rapid microwave digestion method for determination of total organic carbon in soil. Commun. Soil Sci. Plant Anal. 49, 2103–2112. doi: 10.1080/00103624.2018.1495732

[ref2] Brown R. Markewitz D. (2018). Soil heterotrophic respiration: measuring and modeling seasonal variation and silvicultural impacts. For. Ecol. Manag. 430, 594–608. doi: 10.1016/j.foreco.2018.08.018

[ref3] Bunch N. D. Bernot M. J. (2012). Nitrate and ammonium uptake by natural stream sediment microbial communities in response to nutrient enrichment. Res. Microbiol. 163, 137–141. doi: 10.1016/j.resmic.2011.11.004 22155107

[ref4] Burton J. Chen C. Xu Z. Ghadiri H. (2010). Soil microbial biomass, activity and community composition in adjacent native and plantation forests of subtropical Australia. J. Soils Sediments 10, 1267–1277. doi: 10.1007/s11368-010-0238-y

[ref5] Cai W. Xu L. Li M. Sun O. J. He N. (2023). Imbalance of inter-provincial forest carbon sequestration rate from 2010 to 2060 in China and its regulation strategy. J. Geogr. Sci. 33, 3–17. doi: 10.1007/s11442-023-2071-4

[ref6] Chen S. Lu N. Fu B. Wang S. Deng L. Wang L. (2022). Current and future carbon stocks of natural forests in China. For. Ecol. Manag. 511:120137. doi: 10.1016/j.foreco.2022.120137

[ref7] Delgado-Baquerizo M. Maestre F. T. Reich P. B. Jeffries T. C. Gaitan J. J. Encinar D.. (2016). Microbial diversity drives multifunctionality in terrestrial ecosystems. Nat. Commun. 7, 1–8. doi: 10.1038/ncomms10541, PMID: 26817514 PMC4738359

[ref8] Deng J. Zhou Y. Bai X. Luo J. Yin Y. Zhu W. (2019). Soil microbial functional diversity responses to different revegetation types in Baishilazi nature reserve. Pol. J. Environ. Stud. 28, 3675–3686. doi: 10.15244/pjoes/99100

[ref9] Doornbos R. F. van Loon L. C. Bakker P. (2012). Impact of root exudates and plant defense signaling on bacterial communities in the rhizosphere. Agron. Sustain. Develop. 32, 227–243. doi: 10.1007/s13593-011-0028-y

[ref10] Dou X. Yu H. Z. Wang J. Y. Li F. Liu Q. Sun L.. (2023). Effect of prescribed burning on the small-scale spatial heterogeneity of soil microbial biomass in *Pinus koraiensis* and *Quercus mongolica* forests of China. J. For. Res. 34, 609–622. doi: 10.1007/s11676-022-01516-y

[ref11] Feigl V. Ujaczki E. Vaszita E. Molnar M. (2017). Influence of red mud on soil microbial communities: application and comprehensive evaluation of the Biolog Eco plate approach as a tool in soil microbiological studies. Sci. Total Environ. 595, 903–911. doi: 10.1016/j.scitotenv.2017.03.266, PMID: 28432990

[ref12] Fu G. Shen Z. X. Zhang X. Z. Zhou Y. T. (2012). Response of soil microbial biomass to short-term experimental warming in alpine meadow on the Tibetan Plateau. Appl. Soil Ecol. 61, 158–160. doi: 10.1016/j.apsoil.2012.05.002

[ref13] Garland J. Mills A. (1991). Classification and characterization of heterotrophic microbial communities on the basis of patterns of community-level sole-carbon-source utilization. Appl. Environ. Microbiol. 57, 2351–2359. doi: 10.1128/aem.57.8.2351-2359.1991, PMID: 16348543 PMC183575

[ref14] Ge Z. W. Du H. J. Gao Y. L. Qiu W. F. (2018). Analysis on metabolic functions of stored Rice microbial communities by BIOLOG ECO microplates. Front. Microbiol. 9:1375. doi: 10.3389/fmicb.2018.01375, PMID: 30018600 PMC6037723

[ref15] He H. Z. Zhang Z. M. Zhou W. Zhou Y. C. (2022). Soil aggregates, distribution characteristics and organic carbon protection mechanism of *Pinus massoniana* forests of different ages. Pak. J. Bot. 54, 1803–1812. doi: 10.30848/pjb2022-5(11)

[ref16] Heinrich V. Dalagnol R. Cassol H. Rosan T. de Almeida C. Silva J. C.. (2021). Large carbon sink potential of secondary forests in the Brazilian Amazon to mitigate climate change. Nat. Commun. 12, 1–11. doi: 10.1038/s41467-021-22050-1 33741981 PMC7979697

[ref17] Hu X. F. Wu F. Sun X. B. Chen H. P. Yin A. Z. Ji K. S. (2022). Joint analysis of growth and wood property of 38-year-old *Pinus massoniana* from 55 provenances. J. Nanjing Forest. Univ. 46, 203–212. doi: 10.12302/j.issn.1000-2006.202104044

[ref18] Huang Z. C. He X. Zhang C. Zhang M. Y. Wang J. N. Hou Y. Q.. (2023). Microbial communities and functions changed in rhizosphere soil of *Pinus massoniana* provenances with different carbon storage. Front. Microbiol. 14:1264670. doi: 10.3389/fmicb.2023.1264670 38029152 PMC10655096

[ref19] Ibell P. Xu Z. Blumfield T. (2010). Effects of weed control and fertilization on soil carbon and nutrient pools in an exotic pine plantation of subtropical Australia. J. Soils Sediments 10, 1027–1038. doi: 10.1007/s11368-010-0222-6

[ref20] Ji K. S. Xu L. A. Wang D. B. Ni Z. X. Wang Z. R. (2022). Progresses and achievements of genetic improvement on Masson pine (*Pinus massoniana*) in China. J. Nanjing Forest. Univ. 46, 10–22. doi: 10.12303/j.issn.1000-2006.202207020

[ref21] Jiao C. C. Yu G. R. He N. P. Ma A. N. Ge J. P. Hu Z. M. (2016). The spatial pattern of grassland aboveground biomass and its environmental controls in the Eurasian steppe. Acta Geograph. Sin. 71, 781–796. doi: 10.11821/dlxb201605007

[ref22] Kaiser O. Pühler A. Selbitschka W. (2001). Phylogenetic analysis of microbial diversity in the rhizoplane of oilseed rape (*Brassica napus* cv. Westar) employing cultivation-dependent and cultivation-independent approaches. Microb. Ecol. 42, 136–149. doi: 10.1007/s002480000121, PMID: 12024277

[ref23] Labriere N. Davies S. Disney M. Duncanson L. Herold M. Lewis S.. (2023). Toward a forest biomass reference measurement system for remote sensing applications. Glob. Chang. Biol. 29, 827–840. doi: 10.1111/gcb.16497, PMID: 36270799 PMC10099565

[ref24] Li Y. C. Li M. Y. Li C. Liu Z. Z. (2020). Forest aboveground biomass estimation using Landsat 8 and sentinel-1A data with machine learning algorithms. Sci. Rep. 10:9952. doi: 10.1038/s41598-020-67024-3, PMID: 32561836 PMC7305324

[ref25] Li C. Liu X. Meng M. J. Zhai L. Zhang B. Jia Z. H.. (2021). The use of Biolog eco microplates to compare the effects of sulfuric and nitric acid rain on the metabolic functions of soil microbial communities in a subtropical plantation within the Yangtze River Delta region. Catena 198:105039. doi: 10.1016/j.catena.2020.105039

[ref26] Liu J. J. Sui Y. Y. Yu Z. H. Shi Y. Chu H. Y. Jin J.. (2014). High throughput sequencing analysis of biogeographical distribution of bacterial communities in the black soils of Northeast China. Soil Biol. Biochem. 70, 113–122. doi: 10.1016/j.soilbio.2013.12.014

[ref27] Liu X. Zhang B. Zhao W. R. Wang L. Xie D. J. Huo W. T.. (2017). Comparative effects of sulfuric and nitric acid rain on litter decomposition and soil microbial community in subtropical plantation of Yangtze River Delta region. Sci. Total Environ. 601-602, 669–678. doi: 10.1016/j.scitotenv.2017.05.151, PMID: 28577402

[ref28] Lucas Borja M. E. Candel D. Jindo K. Moreno J. L. Andres M. Bastida F. (2012). Soil microbial community structure and activity in monospecific and mixed forest stands, under Mediterranean humid conditions. Plant Soil 354, 359–370. doi: 10.1007/s11104-011-1072-8

[ref29] Mendoza B. Bejar J. Luna D. Osorio M. Jimenez M. Melendez J. R. (2020). Differences in the ratio of soil microbial biomass carbon (MBC) and soil organic carbon (SOC) at various altitudes of Hyperalic Alisol in the Amazon region of Ecuador. F1000Research 9:443. doi: 10.12688/f1000research.22922.1, PMID: 32551098 PMC7281642

[ref30] Mercado L. M. Bellouin N. Sitch S. Boucher O. Huntingford C. Wild M.. (2009). Impact of changes in diffuse radiation on the global land carbon sink. Nature 458, 1014–1017. doi: 10.1038/nature07949, PMID: 19396143

[ref31] Nguyen L. T. T. Osanai Y. Lai K. Anderson I. C. Bange M. P. Tissue D. T.. (2018). Responses of the soil microbial community to nitrogen fertilizer regimes and historical exposure to extreme weather events: flooding or prolonged-drought. Soil Biol. Biochem. 118, 227–236. doi: 10.1016/j.soilbio.2017.12.016

[ref32] Pan P. Han T. Y. Ouyang X. Z. Liu Y. Q. Zang H. Ning J. K.. (2017). Carbon density distribution characteristics and influencing factors in aerially seeded *Pinus massoniana* plantations. Chin. J. Appl. Ecol. 28, 3841–3847. doi: 10.13287/j.1001-9332.201712.001 29696878

[ref33] Rath K. M. Fierer N. Murphy D. V. Rousk J. (2019). Linking bacterial community composition to soil salinity along environmental gradients. ISME J. 13, 836–846. doi: 10.1038/s41396-018-0313-8, PMID: 30446737 PMC6461869

[ref34] Schweinsberg Mickan M. Joergensen R. Mueller T. (2012). Rhizodeposition: its contribution to microbial growth and carbon and nitrogen turnover within the rhizosphere. J. Plant Nutr. Soil Sci. 175, 750–760. doi: 10.1002/jpln.201100300

[ref35] Setia R. Verma S. L. Marschner P. (2012). Measuring microbial biomass carbon by direct extraction - comparison with chloroform fumigation-extraction. Eur. J. Soil Biol. 53, 103–106. doi: 10.1016/j.ejsobi.2012.09.005

[ref36] Terrer C. Jackson R. Prentice I. Keenan T. Kaiser C. Vicca S.. (2019). Nitrogen and phosphorus constrain the CO2 fertilization of global plant biomass. Nat. Clim. Chang. 9:684. doi: 10.1038/s41558-019-0545-2

[ref37] Tyree M. C. Seiler J. R. Maier C. A. (2014). Contrasting genotypes, soil amendments, and their interactive effects on short-term total soil CO2 efflux in a 3-year-old *Pinus taeda* L. plantation. Soil Biol. Biochem. 69, 93–100. doi: 10.1016/j.soilbio.2013.10.050

[ref38] Waldrop M. P. Firestone M. K. (2004). Microbial community utilization of recalcitrant and simple carbon compounds: impact of oak-woodland plant communities. Oecologia 138, 275–284. doi: 10.1007/s00442-003-1419-9, PMID: 14614618

[ref39] Wang L. Chen Z. Shang H. Wang J. Zhang P. Y. (2014). Impact of simulated acid rain on soil microbial community function in Masson pine seedlings. Electron. J. Biotechnol. 17, 199–203. doi: 10.1016/j.ejbt.2014.07.008

[ref40] Wang Y. Zong N. He N. P. Zhang J. J. Tian J. Li L. T. (2018). Soil microbial functional diversity patterns and drivers along an elevation gradient on Qinghai-Tibet, China. Acta Geographica Sinica 38, 5837–5845. doi: 10.5846/stxb201707261343

[ref41] Wei Y. C. Ouyang Z. Y. Miao H. Zheng H. (2009). Exotic *Pinus carbaea* causes soil quality to deteriorate on former abandoned land compared to an indigenous Podocarpus plantation in the tropical forest area of southern China. J. For. Res. 14, 221–228. doi: 10.1007/s10310-009-0130-z

[ref42] Wu L. K. Lin X. M. Lin W. X. (2014). Advances and perspective in research on plant-soil-microbe interactions mediated by root exudates. Chinese J. Plant. Ecol. 38, 298–310. doi: 10.3724/SP.J.1258.2014.00027

[ref43] Xia P. H. Kou Y. Z. Yu L. F. (2015). Carbon metabolic soil microbial community in Caohai karst plateau degraded wetland: a case study in Southwest China. Acta Sci. Circumst. 35, 2549–2555. doi: 10.13671/j.hjkxxb.2014.1010

[ref44] Xia L. Zhao B. Q. Luo T. Xu W. N. Guo T. Xia D. (2022). Microbial functional diversity in rhizosphere and non-rhizosphere soil of different dominant species in a vegetation concrete slope. Biotechnol. Biotechnol. Equip. 36, 379–388. doi: 10.1080/13102818.2022.2082319

[ref45] Yang W. Cai A. D. Wang J. S. Luo Y. Q. Cheng X. L. An S. Q. (2020). Exotic *Spartina alterniflora* Loisel. Invasion significantly shifts soil bacterial communities with the successional gradient of saltmarsh in eastern China. Plant Soil 449, 97–115. doi: 10.1007/s11104-020-04470-y

[ref46] Yao Z. Jiao P. Wu X. Yan Q. Liu X. Hu Y.. (2023). Effect of fire-deposited charcoal on soil organic carbon pools and associated enzyme activities in a recently harvested *Pinus massoniana* plantation subjected to broadcast burning. Huan Jing Ke Xue 44, 4201–4210. doi: 10.13227/j.hjkx.202209081, PMID: 37438317

[ref47] Yao Z. W. Zhang X. D. Wang X. Shu Q. Liu X. M. Wu H. L.. (2023). Functional diversity of soil microorganisms and influencing factors in three typical water-conservation forests in Danjiangkou reservoir area. Forests 14:67. doi: 10.3390/f14010067

[ref48] Zeng W. Xiao Q. (2011). Analysis on carbon content factors of different organs on *Masson pine* in southern China. Central South Forest Invent. Plan. 30, 51–55. doi: 10.16166/j.cnki.cn43-1095.2011.02.013

[ref49] Zhang W. J. Wang J. H. Zhu L. S. Wang J. Mao S. S. Yan X. J.. (2023). New insights into the effects of antibiotics and copper on microbial community diversity and carbon source utilization. Environ. Geochem. Health 45, 4779–4793. doi: 10.1007/s10653-023-01491-1, PMID: 36939996

[ref50] Zhou Y. Sha M. Y. Jin H. Q. Wang L. F. Zhang J. Xu Z. F.. (2023). The expansion of evergreen and deciduous shrubs changed the chemical characteristics and biological community of alpine meadows soil. Eur. J. Soil Biol. 117, 103505–103510. doi: 10.1016/j.ejsobi.2023.103505

[ref51] Zhu P. Chen R. Song Y. Liu G. Chen T. Zhang W. (2015). Effects of land cover conversion on soil properties and soil microbial activity in an alpine meadow on the Tibetan plateau. Environ. Earth Sci. 74, 4523–4533. doi: 10.1007/s12665-015-4509-1

[ref52] Zinn Y. Lal R. Resck D. (2005). Texture and organic carbon relations described by a profile pedotransfer function for Brazilian Cerrado soils. Geoderma 127, 168–173. doi: 10.1016/j.geoderma.2005.02.010

