# Supplementary material for: Analysis on metabolic functions of rhizosphere microbial communities of Pinus massoniana provenances with different carbon storage by Biolog Eco microplates
Source: Front Microbiol. 2024 Mar 6;15:1365111. doi: 10.3389/fmicb.2024.1365111 (PMC10951076; doi:10.3389/fmicb.2024.1365111)
Supplement: Supplementary file 1 [file Data_Sheet_1.docx]

TableS1 Biomass and carbon storages of six *P. massoniana* provenances

| Provenance | height/m  mean | DBH/cm  mean | Biomass/kg  mean | Carbon storages/kg mean |
| --- | --- | --- | --- | --- |
| AY | 18.9 | 22 | 218.71 | 109.42 |
| SM | 17.9 | 25.1 | 285.75 | 142.98 |
| QJ | 16.5 | 19.2 | 153.52 | 76.84 |
| SW | 16.30 | 19.40 | 156.27 | 80.99 |
| HF | 15.3 | 15.4 | 91.87 | 46.00 |
| SX | 14.5 | 15.1 | 86.06 | 43.10 |


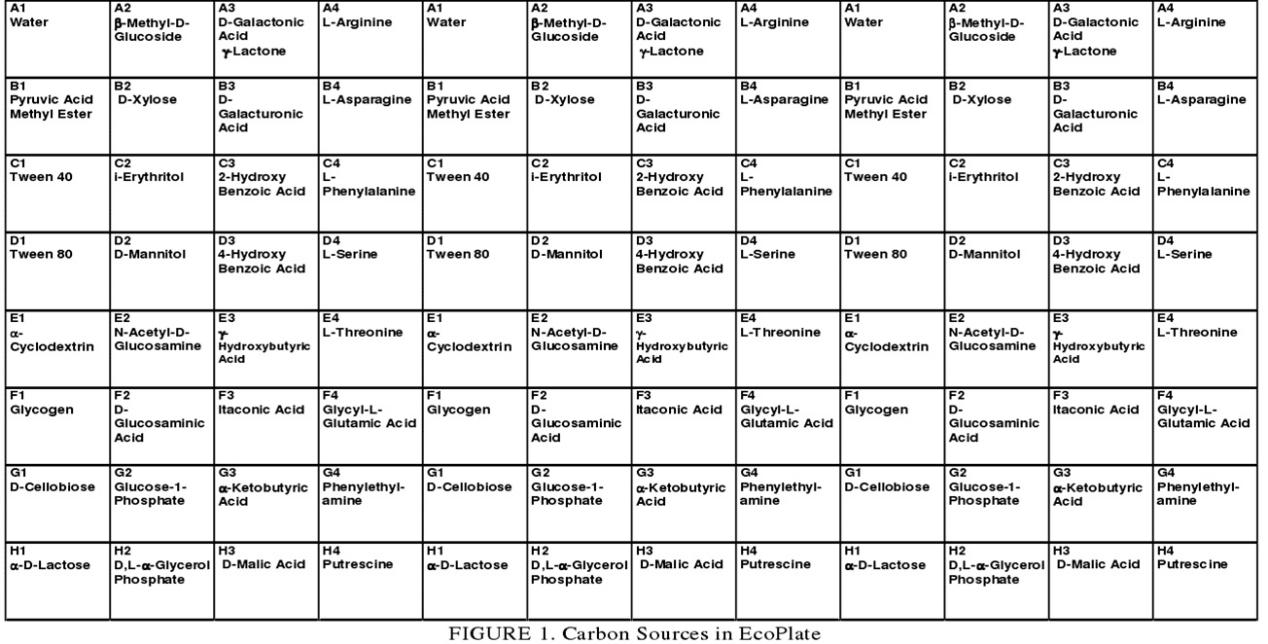


**Figure S1. The distribution of carbon sources on the Biolog Eco** **microplate.**
